# The effect of blood glucose and lipid risk factors on idiopathic sudden sensorineural hearing loss: A two-sample Mendelian randomization study

**DOI:** 10.1016/j.bjorl.2025.101579

**Published:** 2025-05-03

**Authors:** Bang-yu Deng, Yun-xia Zhao, Ji-sheng Liu

**Affiliations:** a The affiliated Suzhou hospital of Nanjing Medical University, Department of Otolaryngology, Jiangsu, China; b Suzhou Jinji Lake Health Service Center, Department of Maternal and Child Health, Jiangsu, China; c First Affiliated Hospital of Soochow University, Department of Otolaryngology, Jiangsu, China

**Keywords:** Idiopathic sudden sensorineural hearing loss, Mendelian randomization, Blood glucose, Lipid metabolism, Triglycerides

## Abstract

**Objective:**

To delve into the potential associations of blood glucose and lipid parameters with Sensorineural Hearing Loss (SHL) and Idiopathic Sudden Hearing Loss (ISHL) through a bidirectional two-sample Mendelian Randomization (MR) analysis, providing insights into the metabolic contributions to hearing loss.

**Methods:**

Genome-Wide Association Studies (GWAS) database from European populations were analyzed to assess the causal effects of blood glucose and lipid risk factors on SHL and ISHL. Single Nucleotide Polymorphisms (SNPs) meeting strict p-value thresholds were used as instrumental variables. MR methods, including Inverse Variance-Weighted (IVW), MR-Egger, and weighted median, were employed. Heterogeneity and pleiotropy were evaluated using Cochran’s *Q* and MR-Egger intercept. Reverse MR analysis assessed the potential impact of SHL and ISHL on metabolic factors.

**Results:**

Triglycerides (TG) exhibited a significant causal effect on SHL (OR = 1.162, 95% CI 1.072–1.260, *p* < 0.01), with findings consistent across MR methods. No other metabolic factors exhibited significant causal effects on SHL. Similarly, no associations were found between any metabolic factors and ISHL. Reverse MR analysis revealed that ISHL may causally influence fasting glucose (OR = 1.001, 95% CI 1.001–1.013, *p* < 0.01) and T2DM (OR = 1.039, 95% CI 1.029–1.049, *p* < 0.01), but SHL showed no significant reverse effects.

**Conclusion:**

TG is a causal risk factor for SHL, whereas ISHL may influence glucose metabolism. These findings underscore the metabolic distinctions between SHL and ISHL and their respective etiologies.

**Level of evidence:**

Level 2.

## Highlights

• TG was a potential risk factor for SHL.
• Increased TG levels significantly raising the risk of SHL.
• Blood glucose and other lipid risk factors did not increase ISSHL risk.

## Introduction

Idiopathic Sudden Sensorineural Hearing Loss (ISSHL) is a common emergency in the department of otolaryngology with an unknown etiology. It is characterized by a sudden decrease in sensorineural hearing loss of ≥30 decibels in at least three consecutive hearing frequencies within a 72-h period. Patients with ISSHL may also experience tinnitus, a feeling of ear congestion, and vertigo.^1^, ^2^ According to the World Health Organization, 2.5 billion people worldwide are expected to experience varying degrees of hearing loss by 2050.^3^, ^4^ Hearing loss has been recognized as one of the leading causes of disability worldwide and the third leading cause of disability-related productivity loss for many years, severely affecting patients' quality of life.^5^, ^6^, ^7^, ^8^ Given the significant impact of ISSHL on individuals and society, researchers have been searching for the complex pathophysiologic mechanisms and underlying causes of ISSHL over the past decades.^9^ However, research on ISSHL has faced great challenges because of the suddenness and unpredictability of this disease.^9^

Abnormal glucose and lipid metabolism has been widely studied and discussed by data scientists as a potential etiologic factor.^10^ Such metabolic abnormalities, particularly diabetes-related hyperglycemia and hyperlipidemia, have been shown to be associated with some chronic diseases such as cardiovascular diseases and diabetic retinopathy.^11^ Aunedeng *et al*^12^ quantified the association between diabetes and blood glucose and heart failure through a meta-analysis of prospective and population-based studies. They therefore found blood glucose and lipid metabolism was associated with the risk of developing heart failure in diabetic patients. However, PARK *et al*^13^ showed that glycemic control had no effect on hearing outcomes in patients with Type 2 Diabetes Mellitus (T2DM) and ISSHL, and systemic corticosteroid could be administered within the allowable range of blood glucose levels. The exact role of abnormal blood glucose and lipid in ISSHL remains unclear.

Mendelian Randomization (MR) is an emerging genetic epidemiological approach that uses genetic variants as an Instrumental Variable (IV) to fully elucidate the causal effect of exposure to risk factors on outcomes.^14^ Since genes are randomly assigned in meiosis, MR generates more meaningful causal conclusions than traditional observational studies by eliminating the interference of other potential confounders and reverse causality.^15^ Zhou *et al* assessed the possible causal relationship between several genetically predicted inflammatory markers and ISSHL by MR analysis. They confirmed that MR analysis provided causal evidence for C-reactive protein as a risk factor for ISSHL.^16^ Building on these insights, this study applies bidirectional two-sample MR analysis to investigate the causal relationships between blood glucose, lipid metabolic factors, and hearing loss. By distinguishing the metabolic contributions to Sensorineural Hearing Loss (SHL) and Idiopathic Sudden Hearing Loss (ISHL), this study aims to provide evidence for targeted interventions and prevention strategies.

## Methods

### Study design

This study used a bidirectional two-sample MR to investigate the causal relationship of ISSHL with blood glucose and lipid risk factors. The MR design was based on three key hypotheses: (1) There is a direct and strong association between genetic variants and exposures; (2) Genetic variants are not associated with potential confounders; and (3) Genetic variants affect outcomes only through exposures and not through other pathways.^17^ All collected data used in this study were publicly available, restricted to a European population, and appropriate ethical approval and patient informed patient were obtained from all previous studies. The publication of this study followed the recommendations of the Statement on Strengthening the Reporting of Observational Studies in Epidemiology Using Mendelian Randomization (STROBE-MR).^18^

### GWAS data source

The data in this study were derived from the Genome-Wide Association Studies (GWAS) database (https://gwas.mrcieu.ac.uk/),^19^ and we selected the data of blood glucose and lipid risk factors including T2DM, fasting glucose, fasting insulin, glycosylated Hemoglobin (HbA1C), Low-Density Lipoprotein Cholesterol (LDL-C), High-Density Lipoprotein Cholesterol (HDL-C), and Triglycerides (TG), as well as samples of SHL and ISHL.

### GWAS samples and screening

In this study, the sample of T2DM included 490,089 cases covering 24,167,560 Single Nucleotide Polymorphisms (SNPs). The sample of fasting glucose included 200,622 cases covering 31,008,728 SNPs. The sample of fasting insulin included 151,013 cases covering 29,664,438 SNPs. The sample of HbA1C included 46,368 cases covering 2,529,804 SNPs. The sample of LDL-C included 343,621 cases covering 19,037,976 SNPs. The sample of HDL-C included 315,133 cases covering 19,051,633 SNPs. The sample of TG included 343,992 cases covering 19,052,580 SNPs. The sample of SHL covered 198,083 cases containing 16,380,454 SNPs. The sample of ISHL included 212,544 cases containing 16,380,454 SNPs.

### Data processing and calibration

This study used a series of rigorous processing steps to ensure the accuracy of data. First, *R* language was applied to initially clean the data and eliminate any possible anomalies or duplicates.^20^ Subsequently, this study used data from a European reference panel of 1000 genomes to eliminate the effects caused by significant linkage disequilibrium among genes. The data were subjected to clustering with a window size of 10,000 kb and the R2 value threshold below 0.001. In this way, the quality of the data was improved to avoid misleading analysis results caused by linkage disequilibrium.

### Mendelian randomization analysis

MR analysis was performed using the TwoSampleMR version 0.5.7 in *R*.^21^ In this analysis, we set a strict criterion of p-value threshold (*p* < 5 × 10^−8^) and selected highly correlated SNPs as IV to assess the causal effects of blood glucose and lipid risk factors on ISSHL.

### Statistical analysis

For MR analysis, SNPs with *p* < 5 × 10^−8^ were selected as IVs in this study. MR-Egger method was used to assess pleiotropy, with a *p*-value < 0.05 indicating the presence of pleiotropy. To evaluate heterogeneity, Cochran's *Q* test was performed for each MR method, with *Q*_pval < 0.05 indicating the presence of heterogeneity.^22^ For assessing pleiotropy, the MR-Egger intercept was calculated, with *p* > 0.05 suggesting the absence of horizontal pleiotropy. These statistical thresholds ensure the robustness of causal inferences by identifying potential violations of MR assumptions.

For ensuring the credibility of the study, three methods Inverse Variance-Weighted (IVW), MR-Egger and weighted median were used to estimate pleiotropy and heterogeneity.^16^ SNPs with *p* < 5 × 10^−8^ were selected as IVs, MR-Egger method was used to assess pleiotropy, with Pval > 0.05 indicating the absence of pleiotropy; Cochran’s *Q* statistic was used to assess heterogeneity, with *Q*_pval > 0.05 indicating the absence of heterogeneity (details in Supplementary Table 1).

In the result validation and consistency test, we required that the positive results were significant at least in the IVW method (*p* < 0.05) to ensure the reliability of the results. Additionally, the estimated effects of the IVW, MR-Egger, and weighted median methods were in the same direction^16^, ^23^ to ensure the consistency and credibility of the conclusions of the study. Through the above exhaustive and rigorous analytical processes, we were able to draw credible conclusions about the relationship between blood glucose and lipid risk factors and hearing loss.

## Results

### Causal analysis of blood glucose and lipid parameters on SHL and ISHL

**Fig. 1:**
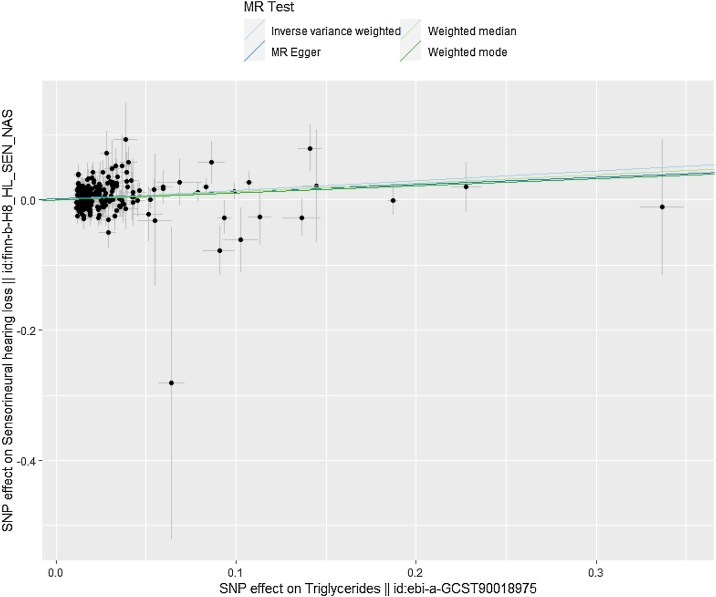
Scatter plot for the association of triglycerides with sensorineural hearing loss (SHL). Data points represent individual SNPs, with regression lines corresponding to different MR methods, visualizing a positive association.

**Fig. 2:**
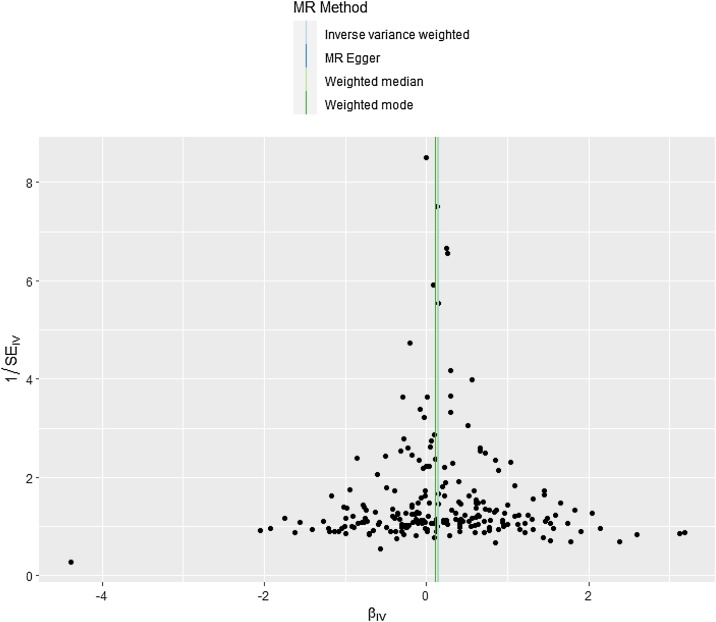
Funnel plot of causal effects of sensorineural hearing loss (SHL)-associated SNPs. The horizontal coordinate represented the effect size of IVW (βiv), the vertical coordinate represented the inverse of the standard error of IVW, and the vertical line represented the estimated effect and standard error of IVW.

This study used a two-sample MR approach to analyze the causal effects of blood glucose and lipid factors on SHL and ISHL. Based on Forest plots and scatter plots revealed a potential causal relationship between TG and SHL, with statistically significant results (*p* < 0.001). We also found consistent trends in IVW, MR-Egger, and weighted median, observing that the probability of developing SHL increased with TG (Fig. S1 and Fig. 1, Supplementary Table 1). The absence of heterogeneity (Cochran's *Q p* = 0.280) and pleiotropy (MR-Egger intercept *p* = 0.377) was confirmed for the association between TG and SHL, validating the consistency of the findings across IVW, MR-Egger, and weighted median methods (Fig. 2, Supplementary Table 2). Collectively, the probability of having SHL increased as TG increased, and there was no heterogeneity or pleiotropy.

### Triglycerides and SHL risk

**Fig. 3:**
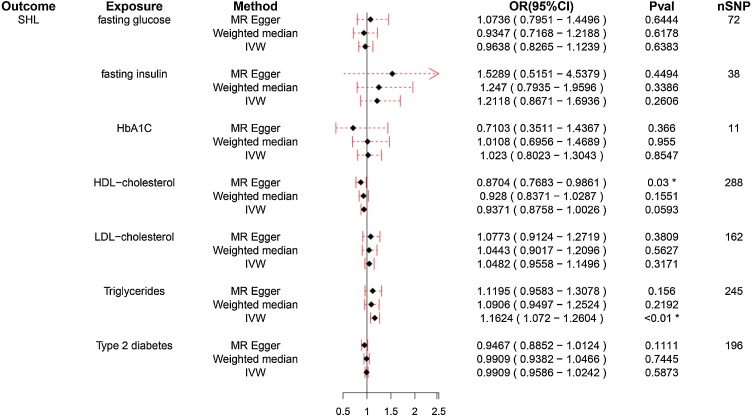
Forest plot for the association of different glucose and lipid metabolism indicators with sensorineural hearing loss (SHL). Forest plot illustrating the causal relationship between metabolic indicators and SHL. TG demonstrates a significant positive association with SHL risk (OR = 1.161), while other markers show no significant effects. The x-axis represents the Odds Ratio (OR) of SHL for each indicator, with OR values greater than 1 suggesting a positive association with increased SHL risk. Points represent the estimated OR for each metabolic indicator, while horizontal lines indicate the 95% Confidence Intervals (95% CI), reflecting the precision of the estimates. A line crossing the value of 1 on the x-axis indicates a lack of significant association. *Indicates that the difference is statistically significant (*p* < 0.05).

**Fig. 4:**
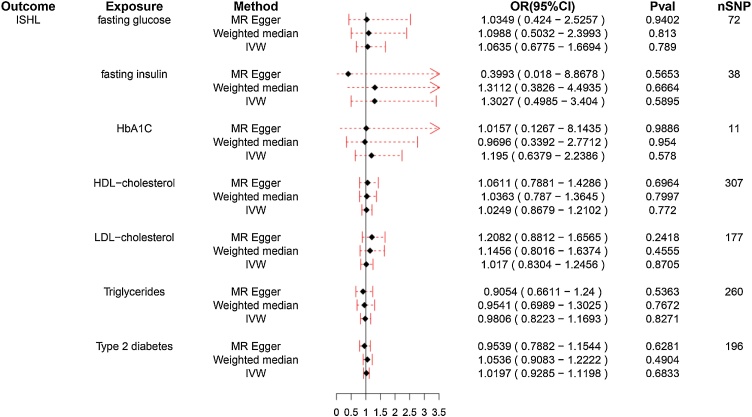
Forest plot for the association of different glucose and lipid metabolism indexes with idiopathic sudden hearing loss (ISHL). Forest plot showing the causal effects of metabolic indicators on ISHL. The indicators include Triglycerides (TG), Low-Density Lipoprotein Cholesterol (LDL-C), High-Density Lipoprotein Cholesterol (HDL-C), fasting glucose, fasting insulin, glycosylated Hemoglobin (HbA1C), and Type 2 Diabetes Mellitus (T2DM). The x-axis represents the Odds Ratios (OR) with 95% Confidence Intervals (95% CI), and the horizontal lines denote the precision of the estimates.

Further this study analyzed the association between different glucose and lipid metabolism indexes (TG, HDL-C, LDL-C, fasting insulin, fasting glucose, HbA1C, T2DM) and SHL/ISHL. As illustrated in Fig. 3, IVW method showed that TG levels were significant positive association with SHL risk (OR = 1.162, 95% CI 1.072–1.260, *p* < 0.01), while other lipid and glucose markers, including HDL-C, LDL-C, fasting insulin, fasting glucose, HbA1C, and T2DM, exhibited no significant causal effect on SHL (Fig. 3, *p* > 0.05). Conversely, Fig. 4 reveals that none of the tested metabolic indicators demonstrated a significant causal relationship with ISHL, further underscoring the specific role of TG in SHL and the absence of significant metabolic contributors to ISHL.

### Reverse causal analysis of SHL and ISHL on blood glucose and lipid metabolic factors

**Fig. 5:**
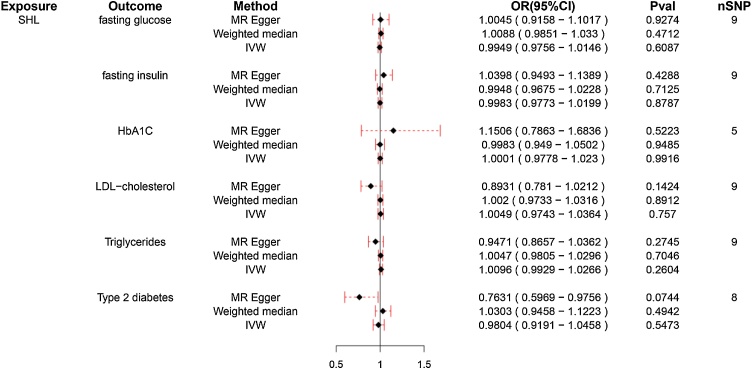
Reverse Mendelian Randomization analysis of Sensorineural Hearing Loss (SHL) on blood glucose and lipid metabolic factors. This figure illustrates the reverse Mendelian Randomization framework assessing the potential causal effects of SHL on blood glucose and lipid metabolism, including Triglycerides (TG), LDL-C, HDL-C, fasting glucose, fasting insulin, glycosylated Hemoglobin (HbA1C), and Type 2 Diabetes Mellitus (T2DM). The horizontal lines represent 95% Confidence Intervals (95% CI).

**Fig. 6:**
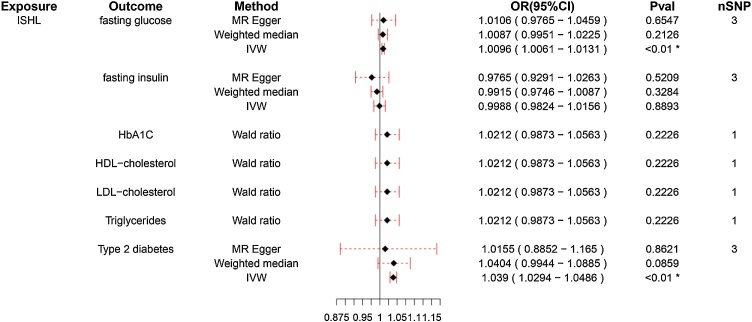
Reverse Mendelian Randomization analysis of Idiopathic Sensorineural Hearing Loss (ISHL) on blood glucose and lipid metabolic factors. The reverse Mendelian Randomization analysis framework investigates the potential influence of ISHL on blood glucose and lipid metabolic markers, including TG, LDL-C, HDL-C, fasting glucose, fasting insulin, HbA1C, and T2DM. The horizontal lines represent 95% Confidence Intervals (95% CI). *Indicates that the difference is statistically significant (*p* < 0.05).

Reverse MR analysis was conducted to explore whether SHL and ISHL exert a causal influence on blood glucose and lipid-related metabolic factors. Findings from Fig. 5 reveal that SHL does not have a significant reverse causal effect on any of the blood glucose or lipid indicators, including TG, LDL-C, HDL-C, fasting glucose, fasting insulin, HbA1c, and T2DM. This suggests no reverse causality from SHL to these metabolic indicators. Conversely, Fig. 6 demonstrates a noteworthy finding regarding ISHL. Using IVW analysis, ISHL shows a potential causal relationship with fasting glucose (OR = 1.001, 95% CI 1.001–1.013, *p* < 0.01) and T2DM (OR = 1.039, 95% CI 1.029–1.049, *p* < 0.01), indicating that the occurrence of ISHL may influence these blood glucose-related metrics.

## Discussion

This study investigated the causal relationships between blood glucose and lipid metabolic factors and two forms of hearing loss ‒ SHL and ISHL ‒ using a bidirectional two-sample MR approach. The findings provide robust evidence that elevated TG levels have a causal relationship with increased SHL risk, supported by consistent results across multiple MR methods and sensitivity analyses. No significant associations were observed between SHL and other metabolic factors, including glucose-related markers and other lipids. In contrast, ISHL did not show any significant metabolic risk factors in the forward MR analysis. However, reverse MR analysis suggested that ISHL might influence fasting glucose and T2DM, revealing potential bidirectional relationships unique to ISHL. These findings underscore the metabolic differences between SHL and ISHL, suggesting TG as a specific risk factor for SHL and a potential role of glucose metabolism in ISHL pathophysiology.

As a form of ISSHL, SHL is characterized by a sudden onset of cochlear or auditory nerve hearing loss over a short period of time without obvious external auditory canal or middle ear diseases.^24^ By contrast, ISHL is more specific in its clinical features and usually has no identifiable cause, and may be accompanied by other symptoms, such as tinnitus, dizziness, sensations of congestion in the ear, or ear fullness. Patients with ISHL may suddenly experience a decrease in hearing early in the morning or upon waking up. Both types of hearing loss have a significant impact on patients' quality of life and psychological well-being.^25^ Blood glucose and lipid metabolism indexes such as LDL-C, HDL-C, TG play a key role in T2DM and nonalcoholic fatty liver disease.^26^ However, Borges MC *et al*.^27^ performed the largest GWAS and a comprehensive MR analysis and their findings did not support the conclusion that the concentration of circulating Polyunsaturated Fatty Acid (PUFA) had a protective role in cardiovascular diseases. Docosahexaenoic acid, a PUFA, has been reported to be associated with an increased risk of some cardiovascular endpoints. But these findings were not subjected to multiple testing adjustments and estimates were inconsistent between different cardiovascular endpoints. Thus, further studies are still needed to confirm these findings.

TG is one of the critical risk factors for cardiovascular and metabolic diseases, and high levels of TG are associated with an increased risk of cardiovascular disease. Maintaining appropriate TG levels is important for maintaining to overall metabolic health.^28^ Selvaraj *et al* revealed the relationship between TG and diabetes.^29^ Specifically, stratified GWAS identified 19 and 315 risk genes significantly associated with TG levels in 21,176 T2DM samples and 402,944 non-T2DM samples in UK Biobank, respectively. They identified a locus located near the HLA-DQB1/DQA2 genes was significantly associated with TG levels in patients with T2DM. Rupali Sharma^30^ included 68 patients with clinically diagnosed SHL between the ages of 20–60 years, and found that total cholesterol and TG levels were significantly associated with the degree of hearing loss (*p* < 0.001). It should be noted that the severity of hearing loss was found to increase significantly with increasing LDL-C levels (*p* < 0.001), whereas HDL levels showed a non-significant negative correlation with the severity of hearing loss. Our study found a positive correlation between SHL risk and TG levels, which is consistent with the study by Rupali Sharma.^30^

Our results also align with previous studies,^31^ which identified associations between lipid metabolism and sudden hearing loss. However, our study extends these findings by including a more comprehensive bidirectional MR approach. Unlike the cited study, we observed no significant reverse causal effects of SHL on metabolic factors, suggesting a one-way relationship between elevated TG levels and SHL risk. Additionally, the novel finding that ISHL causally affects glucose metabolism highlights a distinct metabolic mechanism underlying ISHL, not reported in the prior study. These differences enhance the understanding of metabolic contributions to SHL and ISHL and underscore the importance of distinguishing between the two conditions in research and clinical practice.

The identification of TG as a causal risk factor for SHL has significant clinical implications. TG levels are modifiable through lifestyle interventions, such as dietary adjustments, weight management, and physical activity, as well as pharmacological treatments, including statins, fibrates, and omega-3 fatty acids.^32^, ^33^ Routine lipid profiling in individuals at risk of hearing loss may allow for early identification and intervention, potentially mitigating the progression of SHL. Furthermore, given the robust association between elevated TG and cardiovascular diseases, integrating hearing loss risk assessment into cardiovascular health management could provide dual benefits. Future clinical trials are warranted to evaluate whether TG-lowering therapies directly influence SHL outcomes, thereby offering targeted prevention strategies. The reverse MR findings linking ISHL to glucose metabolism suggest a different clinical approach. ISHL may be an early marker or contributor to dysregulated glucose metabolism, highlighting the need for metabolic screening in ISHL patients. Early detection and management of fasting glucose levels or T2DM in these patients could prevent long-term metabolic complications. These results also open avenues for further research into the shared pathophysiological mechanisms between ISHL and metabolic disorders, such as systemic inflammation or microvascular dysfunction.

This study leverages large-scale GWAS datasets and a robust bidirectional MR design to disentangle the complex relationships between metabolic factors and hearing loss. Unlike prior studies,^31^ the inclusion of reverse MR analyses provides novel evidence for a bidirectional relationship between ISHL and glucose metabolism, offering new perspectives on the etiology and management of ISHL. Additionally, the identification of TG as a significant risk factor for SHL underscores the importance of lipid profile monitoring in at-risk individuals. Regular screening for TG levels in patients with a family history of hearing loss or other risk factors could facilitate early interventions, including dietary modifications, increased physical activity, and pharmacological therapies such as fibrates and omega-3 fatty acids. Furthermore, integrating TG management into cardiovascular health initiatives could address dual risks, given the shared pathways between SHL and cardiovascular diseases. For patients with ISHL, the reverse MR findings suggest a potential role in glucose dysregulation, highlighting the need for fasting glucose and T2DM screening in these patients. Future clinical trials are warranted to determine whether interventions targeting TG reduction or glucose stabilization improve hearing outcomes and reduce long-term metabolic risks.

Despite its strengths, this study has some limitations. First, the subjects who participated in the GWAS were limited to the European region with big data, and it remains highly questionable whether the same results can be generalized to other ethnicities. Second, the lack of stratified data such as sex and age in the existing aggregated statistics set prevented us from conducting a comprehensive and more refined analysis. More detailed and publicly available stratified data in the future will help to further clarify new MR analyses. Finally, the relatively small sample size of the GWAS for TG may have led to some bias in the results. Therefore, caution should be exercised in interpreting the negative results of TG for ISSHL risk.

## Conclusion

This study provides robust evidence for a causal relationship between elevated TG levels and an increased risk of SHL, emphasizing the need to manage triglyceride levels to reduce SHL risk. In contrast, no significant metabolic risk factors were identified for ISHL in forward MR analysis. However, reverse MR analysis revealed that ISHL may influence glucose metabolism, highlighting a potential bidirectional relationship. These findings contribute to a deeper understanding of the metabolic mechanisms underlying SHL and ISHL, offering new perspectives for targeted prevention and treatment strategies. Further research is required to confirm these associations and explore their clinical implications across diverse populations.

## CRediT authorship contribution statement

Study concept and design: BYD, JSL; Analysis and interpretation of data: BYD, YXZ; Drafting of the manuscript: BYD, JSL; Critical revision of the manuscript for important intellectual content: BYD, JSL; Statistical analysis: YXZ, JSL; Study supervision: all authors; all authors have read and approved the manuscript.

## Funding

This study did not receive any funding in any form.

## Data availability statement

The data used to support the findings of this study are available from the corresponding author upon request.

## Declaration of competing interest

The authors declare no conflicts of interest.
