# Supplementary material for: The effect of blood glucose and lipid risk factors on idiopathic sudden sensorineural hearing loss: A two-sample Mendelian randomization study
Source: Braz J Otorhinolaryngol. 2025 May 3;91(5):101579. doi: 10.1016/j.bjorl.2025.101579 (PMC12104699; doi:10.1016/j.bjorl.2025.101579)

BJORL-D-24-00128_Supplementary Material

**Supplementary Table 1 Overview of three methods used for Mendelian randomization analysis of idiopathic sudden sensorineural hearing loss**

| Outcomes | Exposures | nSNPs | IVW | | MR Egger | | Weighted median | |
| --- | --- | --- | --- | --- | --- | --- | --- | --- |
|  |  |  | OR（95%CI） | p | OR（95%CI） | p | OR（95%CI） | p |
| ISHL | Type 2 diabetes | 171 | 1.025(0.927-1.134) | 0.629 | 0.937(0.766-1.145) | 0.525 | 1.045(0.888-1.231) | 0.595 |
|  | fasting glucose | 63 | 1.016(0.639-1.616) | 0.946 | 0.804(0.349-1.854) | 0.611 | 1.103(0.549-2.216) | 0.783 |
|  | fasting insulin | 38 | 1.356(0.512-3.596) | 0.540 | 0.333(0.015-7.492) | 0.494 | 1.280(0.325-5.042) | 0.724 |
|  | HbA1C | 11 | 1.195(0.525-2.722) | 0.671 | 1.016(0.127-8.144) | 0.989 | 0.970(0.319-2.944) | 0.956 |
|  | LDL-C | 151 | 1.175(0.978-1.411) | 0.086 | 1.317(1.038-1.672) | 0.025 | 1.368(1.015-1.842 | 0.040 |
|  | HDL-C | 255 | 0.986(0.840-1.158) | 0.865 | 1.007(0.798-1.271) | 0.952 | 1.023(0.792-1.320) | 0.864 |
|  | TG | 221 | 1.002(0.832-1.208) | 0.982 | 0.999(0.760-1.315) | 0.997 | 1.050(0.756-1.459) | 0.771 |
| SHL | Type 2 diabetes | 170 | 0.994(0.959-1.029) | 0.719 | 0.947(0.883-1.015) | 0.126 | 0.988(0.935-1.045) | 0.677 |
|  | fasting glucose | 63 | 0.956(0.818-1.118) | 0.576 | 0.964(0.728-1.277) | 0.797 | 0.988(0.766-1.274) | 0.926 |
|  | fasting insulin | 38 | 1.225(0.874-1.715) | 0.238 | 1.458(0.492-4.324) | 0.501 | 1.212(0.776-1.893) | 0.398 |
|  | HbA1C | 11 | 1.023(0.775-1.350) | 0.873 | 0.710(0.351-1.437) | 0.366 | 1.011(0.695-1.469) | 0.955 |
|  | LDL-C | 149 | 0.992(0.928-1.060) | 0.807 | 0.967(0.887-1.054) | 0.449 | 0.957(0.862-1.063) | 0.413 |
|  | HDL-C | 253 | 0.963(0.911-1.018) | 0.188 | 0.978(0.902-1.060) | 0.582 | 1.042(0.948-1.145) | 0.395 |
|  | TG | 218 | 1.161(1.081-1.246) | <0.001 | 1.116(0.998-1.248) | 0.056 | 1.138(1.017-1.274) | 0.025 |

Abbreviations: ISHL, idiopathic sudden hearing loss; SHL, sensorineural hearing loss; HbA1C, glycosylated hemoglobin; LDL-C, low-density lipoprotein cholesterol; HDL-C, high-density lipoprotein cholesterol; TG, triglycerides; IVW, inverse variance-weighted.

**Supplementary Table 2** Mendelian randomization analysis of genetic associations between different exposures and idiopathic sudden sensorineural hearing loss.

| Outcomes | Exposures | Heterogeneity | | Pleiotropy | |
| --- | --- | --- | --- | --- | --- |
|  |  | Q | Q_pval | intercept | pval |
| ISHL | Type 2 diabetes | 186.289 | 0.172 | 0.008 | 0.311 |
|  | fasting glucose | 56.448 | 0.641 | 0.007 | 0.511 |
|  | fasting insulin | 45.154 | 0.141 | 0.024 | 0.358 |
|  | HbA1C | 5.787 | 0.761 | 0.006 | 0.871 |
|  | LDL-C | 136.623 | 0.758 | <0.001 | 0.141 |
|  | HDL-C | 223.155 | 0.912 | <0.001 | 0.806 |
|  | TG | 207.553 | 0.700 | <0.001 | 0.979 |
| SHL | Type 2 diabetes | 195.603 | 0.071 | 0.004 | 0.119 |
|  | fasting glucose | 50.283 | 0.835 | <0.001 | 0.950 |
|  | fasting insulin | 48.414 | 0.081 | <0.001 | 0.742 |
|  | HbA1C | 6.447 | 0.694 | 0.014 | 0.298 |
|  | LDL-C | 169.674 | 0.097 | 0.002 | 0.395 |
|  | HDL-C | 265.156 | 0.258 | <0.001 | 0.658 |
|  | TG | 227.645 | 0.280 | 0.002 | 0.377 |

Abbreviations: ISHL, idiopathic sudden hearing loss; SHL, sensorineural hearing loss; HbA1C, glycosylated hemoglobin; LDL-C, low-density lipoprotein cholesterol; HDL-C, high-density lipoprotein cholesterol; TG, triglycerides

**Figure S1 Forest plot of causal effects of sensorineural hearing loss (SHL)-associated SNPs.** The Random Forest model of SNPs was established, with the scale representing representing predictive power and the red scale indicating effects in the positive direction.


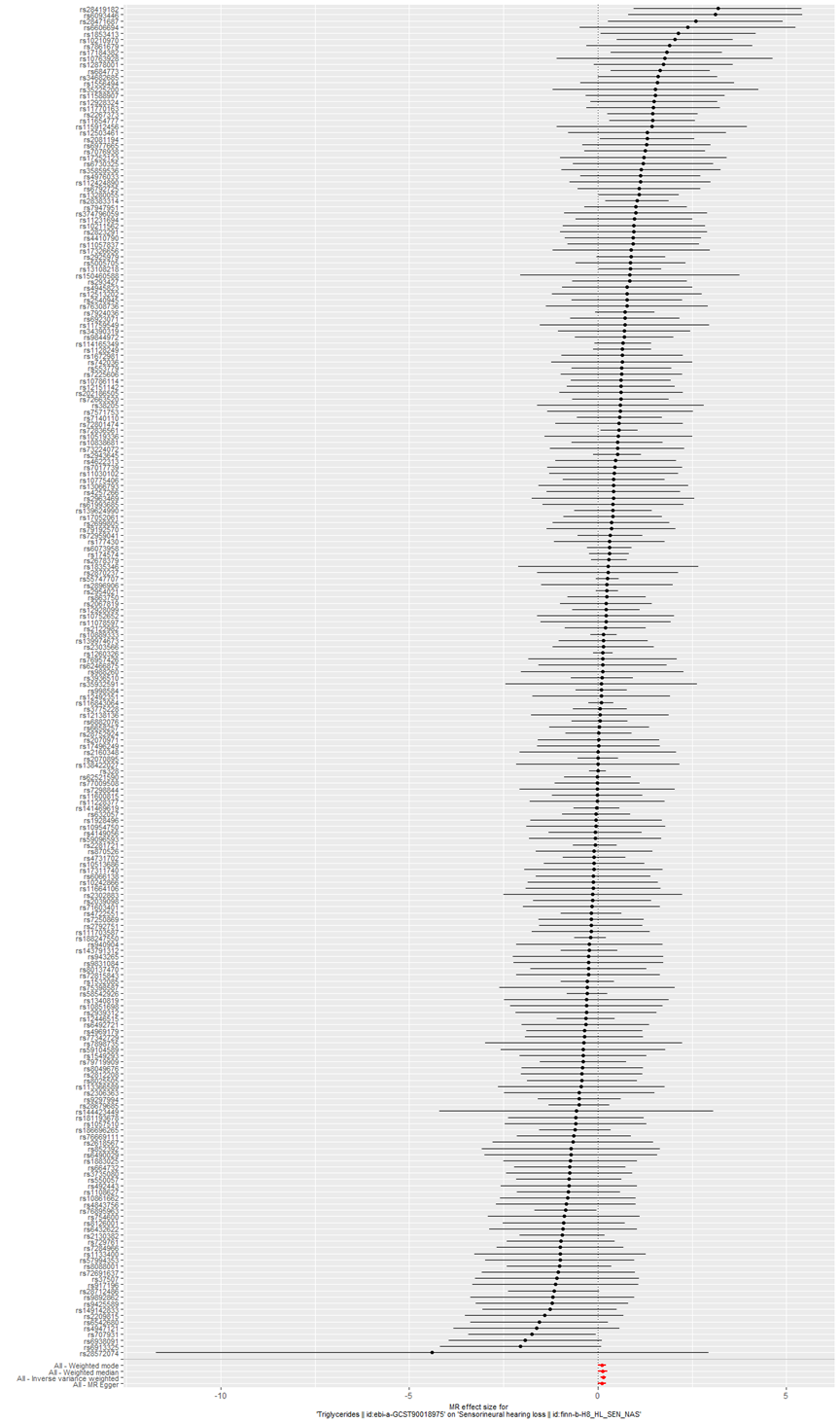

Supplement: Supplementary file 1 [file mmc1.doc]
